# Supplementary material for: DHHC21 is a STIM1 protein S-acyltransferase that modulates immune function in vivo
Source: bioRxiv. 2025 Oct 22:2025.10.09.681383. Preprint. [Version 2] doi: 10.1101/2025.10.09.681383 (PMC12632593; doi:10.1101/2025.10.09.681383)

bioRxiv preprint doi: https://doi.org/10.1101/2025.10.09.681383; this version posted October 22, 2025. The copyright holder for this preprint (which was not certified by peer review) is the author/funder, who has granted bioRxiv a license to display the preprint in perpetuity. It is made available under aCC-BY 4.0 International license.

i

Supplementary Figure 1: Endogenous DHHC21 colocalizes with STIM1 in HEK293 WT cells. Confocal (A and C) and STED (B and C) images of HEK293 WT cells untreated (A and B)

bioRxiv preprint doi: https://doi.org/10.1101/2025.10.09.681383; this version posted October 22, 2025. The copyright holder for this preprint (which was not certified by peer review) is the author/funder, who has granted bioRxiv a license to display the preprint in perpetuity. It is made available under aCC-BY 4.0 International license.

or treated (C and D) with 10 μM thapsigargin (TG). The boxes in Panels A and C were used as the imaging area for STED imaging in Panels B and D. Green represents DHHC21, and red represents STIM1. E) Colocalization was quantified using Pearson's correlation values obtained from cells. Statistical significance between the groups was calculated using Student's t-test. Error bars indicate S.D. Pearson's correlation between DHHC21 and STIM1 obtained from (E) using the JACoP plugin was analyzed using a Student's t-test. Error bars indicate S.D.

bioRxiv preprint doi: https://doi.org/10.1101/2025.10.09.681383; this version posted October 22, 2025. The copyright holder for this preprint (which was not certified by peer review) is the author/funder, who has granted bioRxiv a license to display the preprint in perpetuity. It is made available under aCC-BY 4.0 International license.

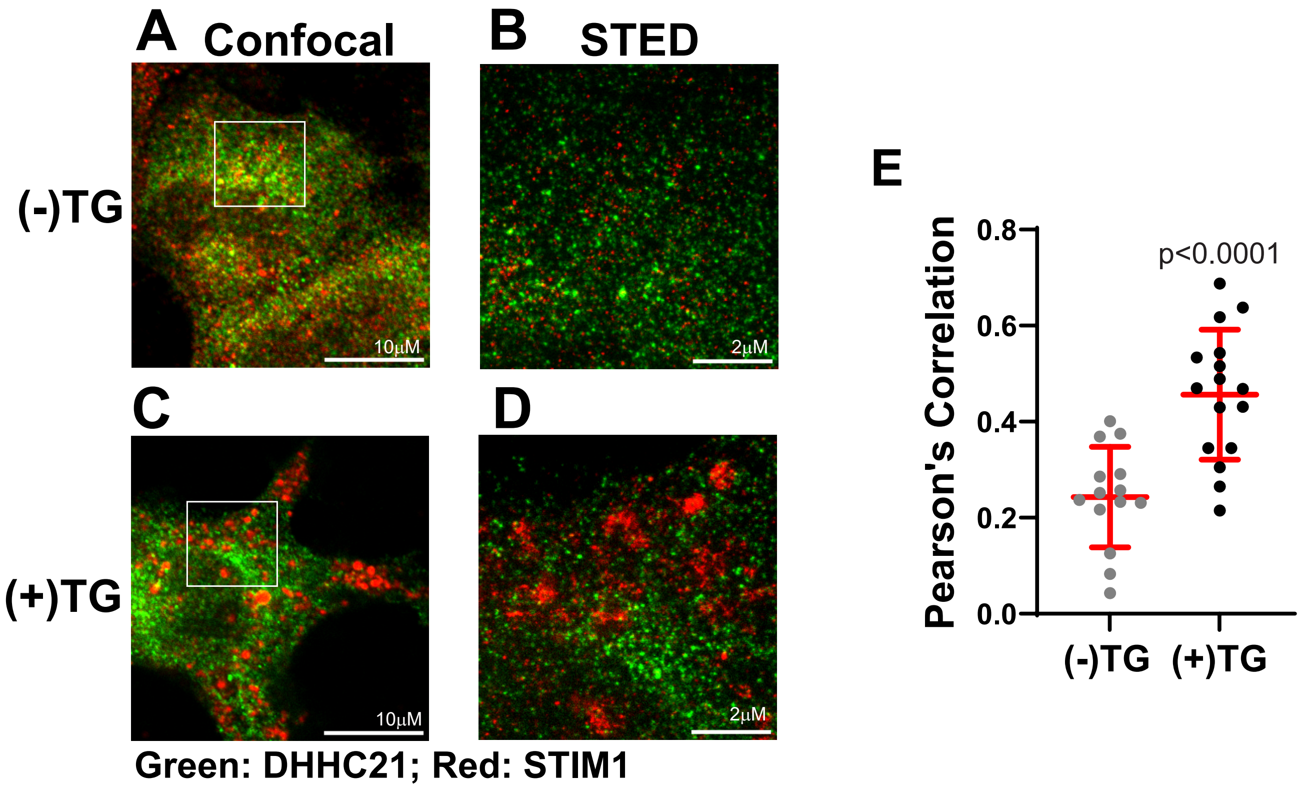

Supplement: Supplement 1 [file NIHPP2025.10.09.681383v2-supplement-1.pdf]
